# Naval Rank

**Published:** 1871-05

**Authors:** 


### Naval Rank.

There may be some young physicians who will be glad to learn how the question of *Rank* in th United States Navy was finally settled. The rank presented below was conferred by Act of Congress, approved March 3, 1871:—

| TITLES. | RELATIVE RANK OF |
|---|---|
| Surgeon-General (Chief of Bureau) | Commodore. |
| Medical Directors | Captain. |
| Medical Inspectors | Commander. |
| Surgeons | Lieut.-Com. or Lieut. |
| Passed Assistant Surgeons | Lieut. or Master. |
| Assistant Surgeons | Master or Ensign. |

